# 

**Published:** 1918-03-16

**Authors:** 


### Manager's Notices.

**All letters on business matters must be addressed to The Manager, "The Hospital," 28 and 29 Southampton Street, London, W.C. 2.**

RATES OF SUBSCRIPTION (payable in advance).

UNITED KINGDOM.

| | | |
|---|---|---|
| Three Months | (including Postage) ... ... ... ... | 2s. 0d. |
| Six Months | do. ... ... ... ... | 4s. 0d. |
| Twelve Months | do. ... ... ... ... | 6s. 6d. |

FOREIGN AND COLONIAL.

| | | |
|---|---|---|
| Three Months | (including Postage) ... ... ... ... | 2s. 6d. |
| Six Months | do. ... ... ... ... | 5s. 0d. |
| Twelve Months | do. ... ... ... ... | 8s. 8d. |

**Special Rates** will be quoted to those institutions that agree to send all their public notices to THE HOSPITAL, so as to make it a file of such announcements for ready reference.

**Subscriptions,** which may commence at any time, are payable in advance. Cheques and Post-Office Orders should be crossed London County and Westminster Bank, Covent Garden Branch, and made payable to the Manager of THE HOSPITAL.

### Gifts and Bequests.

Under the will of Miss Hannah Hewetson, of Ware, Devon, the Cumberland Infirmary benefits to the extent of £333.

Miss Eliza Wellock, of Northenden, Cheshire, after some small bequests, left the residue of her estate of £11,528 to the Manchester and Salford Blind Aid Society.

The Royal Dental Hospital, Leicester Square, has received a gift of Canada 3½ per cent. Registered Stock to the value of £500 from the executors of the late Mrs. Gore-Lloyd.

Under the will of the late Mr. James McCandlish, of Liversidge, the Dewsbury and District Infirmary has been left a legacy of £250, and part of the residue of the estate.

Mrs. Crossley, mother of Mr. C. W. Crossley, president of the Royal Halifax Infirmary, has promised the institution £1,000 to endow a bed in memory of her husband, and her son-in-law, Mr. Marcell Smith, who were lost in the torpedoed *Persia* early in 1916.

By a gift of £1,000, the sons and daughters of the late Colonel and Mrs. James Clifton Brown have endowed in memory of their father and mother a bed in the Hahnemann Ward of the London Homœopathic Hospital, Great Ormond Street, W.C.

The late Mr. Archibald Carmichael, of Perth, has left the residue of his estate, amounting to a sum of about £12,000, for the advancement of the work on Aberdeen University's medical side.

Mr. J. H. Straker, of Corbridge-on-Tyne, has purchased Prior House, Corbridge, and proposes to present it, equipped and furnished as a hospital, to the trustees of the Corbridge Cottage Hospital, as a memorial to the late Mrs. Straker.

### Matrons' and Sisters' Appointments.

Cottage Hospital, Moreton Hampstead, Devon.—Miss M. Lamble has been appointed nurse-matron. She was trained at the North Staffordshire Infirmary and Eye Hospital, Stoke-on-Trent, has been sister at the Liverpool Sanatorium, Frodsham, Cheshire, and at the Royal Berkshire Hospital, Reading, and matron of the Falmouth Hospital.

County and City of Perth Royal Infirmary, Perth.—Miss Emily Godfrey has been appointed night superintendent. She was trained at the York County Hospital, where she has been staff nurse and ward sister.

Hertford County Hospital, Hertford.—Miss Helen White has been appointed sister. She was trained at the Royal West Sussex Hospital, Chichester, and has been sister at the Guest Hospital, Dudley, and at Harrogate Infirmary.

John Coupland Hospital, Gainsborough.—Miss Mary W. Dickson has been appointed matron. She was trained at the Durham County Hospital, where she was later night sister, day sister of the women's and children's wards, theatre sister, senior sister of the men's medical and surgical wards, assistant matron and acting matron.

Royal Victoria Hospital, Boscombe.—Miss Kathleen Fitz-Patrick has been appointed theatre sister. She was trained at the York County Hospital, and has since been theatre sister at the Beckett Hospital, Barnsley.

St. Mary Islington Infirmary, Highgate Hill.—Miss Kate Eleanor Palframan has been appointed assistant matron. She was trained at St. Pancras North Infirmary, has been nurse-matron at Girton College, temporary assistant matron at Islington Infirmary, and assistant matron at Mile End Military Hospital.

Sunderland Royal Infirmary.—Miss Elsie Cockerill and Miss A. M. Taylor have been appointed sisters. They were trained at the York County Hospital, where they have since been staff nurses.

**NOTE.—Particulars of Appointments for insertion in this column will be welcomed.**

### ST. THOMAS'S HOSPITAL WAR RECORD.

The war record of St. Thomas's Hospital shows that up to March 6 there were serving 1,032 past and present students, and that forty-eight were killed in action or had died on service. The honours gained were one V.C., one G.C.M.G., one K.C.B., three K.C.M.G.'s, four C.B.'s, fifteen C.M.G.'s, two M.V.O.'s, twenty-three D.S.O.'s, thirty-nine M.C.'s, and three bars to M.C., two C.I.E.'s, one G.B.E., four O.B.E.'s, one M.B.E., one C.B.E. For valuable service 132 men were mentioned in dispatches (188 times).

The foreign honours include eight French, three Belgian, six Egyptian, and seven Serbian. Five Orders of the Hospital of St. John of Jerusalem were also received.

### Newsvendors' Benevolent and Provident Institution.

There being no annual Festival Dinner during the war on behalf of the Newsvendors' Benevolent and Provident Institution, an appeal has been issued by the trustees of the charity to meet present difficulties in providing income.

### Nurses' Memorial Service.

### St. Paul's Cathedral, Wednesday, April 10.

APPLICATIONS FOR SEATS SHOULD BE MADE AS FOLLOWS:

All **Nurses and V.A.D.s serving in Military Hospitals** to apply through their Matrons to the Matron-in-Chief, Q.A.I.M.N.S., Adastral House, Victoria Embankment.

All **Nurses and V.A.D.s serving in Territorial Hospitals** to apply through their principal Matrons to Miss Sidney Browne, R.R.C., War Office, 80 Pall Mall, S.W.

All the **Overseas Nurses** to apply through their Matrons to their Matrons-in-Chief.

All **trained Nurses working in Red Cross or Auxiliary Hospitals** to apply through their Matrons to their Matron-in-Chief, Miss Swift, R.R.C., 83 Pall Mall, S.W.

All **V.A.D.s serving in Red Cross and Auxiliary Hospitals** to apply through their Matrons to Lady Ampthill and Lady Perrott, Devonshire House, Piccadilly.

All **Civilian Nurses and all relatives of Nurses who have fallen in the War** to apply to the Organizer of the Service the Rev. A. Lombardini, Church of St. Elizabeth, Kensington, W., to whom all inquiries should be addressed. (See paragraph under "Round the Hospitals," page 508.)

### Annual Meetings.

Italian Hospital, Queen Square, W.C.—Wednesday, March 20, at 3 p.m., His Excellency the Italian Ambassador (President) in the chair.

Poplar Hospital for Accidents, Poplar, E. 14.—Tuesday, March 19, at 3.30 p.m.

Royal Dental Hospital of London, Leicester Square, W.C. 2.—Tuesday, March 19, at 3 p.m. The Right Rev. the Lord Bishop of London will preside and deliver an address.

